# Risk factors and treatment outcome of smear positive pulmonary tuberculosis patients: A five-year study in the North of Iran

**DOI:** 10.22088/cjim.15.2.347

**Published:** 2024

**Authors:** Roghieh Golsha, Mahdi Mazandarani, Ahmad Sohrabi, Hesamaddin Shirzad-Aski, Hamidreza Kamalinia, Atefeh Rezaeifar, Mandana Fattahi

**Affiliations:** 1 Infectious Diseases Research Center, Golestan University of Medical Sciences, Gorgan, Iran; 2 Tuberculosis Laboratory of Health care Center, Golestan University of Medical Sciences, Gorgan, Iran; 3 Isfahan Endocrine and Metabolism Research Center, Isfahan University of Medical Sciences, Isfahan, Iran

**Keywords:** Tuberculosis, Risk factor, Poverty, Socioeconomic status, General practitioners training.

## Abstract

**Background:**

It is essential to constantly review the risk factors and treatment outcomes of tuberculosis (TB). This study evaluated some important risk factors of TB over five years.

**Methods:**

Between 2013 and 2018, all available information and possible risk factors related to TB patients were analyzed from the TB registry program of the health district of Gorgan, Iran.

**Results:**

Among 349 TB patients, 194 (55.59%) were males and 167 (47.85%) had at least a comorbidity. The death rate was higher in the age group more than 65 years (p < 0.001), the low-educated group (P = 0.012), and patients with underlying diseases, especially diabetes (p < 0.001). In total, univariate and multivariate statistical analyzes showed that having comorbidity (OR = 4.34; 95% CI 1.49 – 13.49), as well as, being jobless (OR = 3.07; 95% CI 1.19 – 8.59) were the main factors influencing the adverse events.

**Conclusion:**

According to the study, aging, underlying diseases, and cultural poverty include a higher share of the main risk factors for active TB and/or treatment outcomes. By considering these risk factors and training the medical staff continually, we can reduce the time of TB diagnosis, and prevent it from spreading.

The causative agent of tuberculosis (TB) is *Mycobacterium tuberculosis* (MTB), a bacterium that has been associated with humans for centuries (1). The World Health Organization (WHO) has been carefully planning to reduce the disease since the 1990s. In this regard, WHO set “STOP TB” and “END TB by 2030” programs to finally eliminate TB as a public health problem by 2050 (1–3). Despite the extensive efforts of organizations and governments to control TB, this disease remains a significant problem in the society, and although its reduction has been observed in global statistics, it has been slow (only 1.5% per year) and is far from the reduction rate predicted by WHO (4.5% per year by 2020) (4, 5). This issue demonstrates its importance and indicates the need for regular surveys in each region. One of the most important ways to prevent tuberculosis is early diagnosis and timely treatment of TB patients. However, we still need to improve the diagnosis and management systems and fully understand the disease risk factors, to achieve the WHO targets (5, 6). Several environmental and individual risk factors are involved in the emergence, spread, and the persistence of this disease, including age, sex, comorbidities, education, occupation, nutrition, smoking, and alcohol status, as well as lifestyle and other socio-economic determinants. Similar to other infectious diseases, some factors play a minor role and others have a strong involvement in the incidence and severity of TB (7-12). 

Updating this information can be effective in controlling and reducing the burden of disease and can introduce and differentiate the minor and major risk factors of it (4, 13). Thus, the purpose of this study was to update the impact of any factor affecting the diagnosis and treatment outcome of TB in patients in Gorgan, North of Iran. The aims can demonstrate how these risk factors can affect the disease and its treatment, and help in the development of TB prevention, control, and treatment programs in the northern Iran, which can be extended to other similar areas as well.

## Methods

**Study Design:** The information on smear-positive TB patients was obtained in a retrospective cross-sectional study, between September 2013 and August 2018, in Gorgan, Iran. We only use the information of smear-positive TB patients to make sure about their active disease. The Ethics Committee of the University approved the study protocol (ethical approval code: IR. GOUMS_9282523276). All experiments were performed following relevant guidelines of the Declaration of Helsinki principles.

Researchers collected the information (shown in Tables 1 and 2) of the selected patients using local electronic databases, archives, and documents. The incomplete information in the files was the exclusion criteria. During the collection of TB patients’ data, due to the retrospective nature of the study, there was no valid information of Human Immunodeficiency Virus (HIV) infection in most of the cases; therefore, this item is not in the comorbidities category.

**Definition of Variables:** As a treatment outcome, a "cured" (pulmonary TB) patient has followed the WHO definition and is defined as sputum culture-negative in the last month of treatment and on at least one previous occasion. The health system delay was defined as the time between a patient's referral with symptoms and diagnosis of TB in a hospital or other healthcare facility (14).

**Data analysis:** R software Version 4.1.0 was used to evaluate all variables. At first, patient information became anonymous. The descriptive information of patients was reported as mean and standard deviation, or percentages and numbers. To analyze the variables, we used the chi-square (χ2) test along with Fisher's correction where appropriate, and a p-value of less than 0.05 was considered statistically significant.

Based on the grouping of patients according to their outcome, odds ratios (ORs) of variables were calculated using univariate and multivariate logistic regression. Accordingly, the cured patients were classified under the successful treatment category, while patients who did not respond to treatment, as well as those who died, were classified under the unsuccessful treatment category. Any variable that was an important variable related to this disease or was significant in the initial analysis was selected for univariate and multivariate analysis, if there was enough data for analysis in each group (successful/unsuccessful).

## Results

During the study, the information of 349 TB patients were recruited (mean age = 50.84, median = 50.0, range 1 - 95 years). Almost half of the patients (47.85%) had at least one comorbidity. Basic demographic and other information of the participants are presented in table 1.

Among the patients, the most common complaint of clinical symptoms was cough (63.32%). Among the various centers that identified these patients, the most location was healthcare facilities (including Urban Health Centers, Rural Health Centers, and Rural Health House) with a rate of 35.82% (Table 2). 

Tables 1 and 2 also show the difference between the variables based on the three treatment outcome groups, cure, failure, and death. The death rate was higher in men (8.76%) than in women (5.80%), but this difference was not significant (P = 0.399). The mean age varied significantly based on the outcome. In this regard, the mean age of the cured and treatment failure subjects were 48.73 years (median = 48) and 49.41 years (median = 45), respectively, whilst the mean age of the patients with the outcome of death was 76.42 years (median = 80). Table 1 shows the details of age group differences. The number of individuals with death or treatment failure outcomes was significantly higher in the low-educated group (P = 0.012). As expected, death was higher in the group of patients with underlying disease, especially DM (p < 0.001).

Based on success in treatment’s outcome, 301 (86.25%) patients had successful treatment and unfortunately, 48 (13.75) patients had an unsuccessful outcome. Moreover, the result showed that a lower level of education can increase the rate of adverse events (OR = 3; 95% CI = 1.25 - 8.93, P = 0.024). The comorbidities (OR = 5.85; 95% CI = 2.85 - 13.2, p < 0.001) and DM (OR = 3.19; 95% CI = 1.69 - 6.01, p < 0.001) were also factors influencing the unsuccessful outcome (Table 3). Another factor that increased the chances of patient’s death was the lack of a real job among patients, which could significantly increase four times the rate of unsuccessful treatment in an unemployed patient with TB (OR = 4.07; 95% CI = 1.93- 9.65, p < 0.001). Table 3 shows the results of the univariate model analysis. According to the first step of the results, the multivariate model assessed any interaction between unsuccessful treatment outcomes (death or failure) and the potential predictor variables (Table 4). Having comorbidity (OR = 4.34; 95% CI 1.49– 13.49), as well as not having a job (OR = 3.07; 95% CI 1.19– 8.59) were the main factors influencing the adverse events. 

**Table 1:** Patients’ characteristics stratified by the treatment outcome

**Characteristic**	**Overall, N = 349**	**Cured, N= 301**	**Failure, N= 22**	**Death, N= 26**	**P-value** ^*^
**Sex, n (%)**					0.399
Male	194 (55.59)	163 (54.15)	14 (63.64)	17 (65.38)	
Female	155 (44.41)	138 (45.85)	8 (36.36)	9 (34.62)	
**Age**, Mean (SD)	50.84 (20.91)	48.73 (20.06)	49.41 (18.62)	76.42 (15.32)	**< 0.001**
**Age Category, n (%)**					**< 0.001**
< 65	232 (66.48)	212 (70.43)	16 (72.73)	4 (15.38)	
< 25	39 (11.17)	38 (12.62)	1 (4.55)	0 (0.00)	
25 - 45	111 (31.81)	99 (32.89)	10 (45.45)	2 (7.69)	
46 - 64	82 (23.50)	75 (24.92)	5 (22.73)	2 (7.69)	
>= 65	117 (33.52)	89 (29.57)	6 (27.27)	22 (84.62)	
**Education, n (%)**					**0.012**
High Education	83 (23.78)	78 (25.91)	5 (22.73)	0 (0.00)	
Low Education	266 (76.22)	223 (74.09)	17 (77.27)	26 (100.00)	
**Drug use, n (%)**					**< 0.001**
No	247 (70.77)	221 (73.42)	16 (72.73)	10 (38.46)	
Yes	102 (29.23)	80 (26.58)	6 (27.27)	16 (61.54)	
**TB** ^1^ ** History, n (%)**					0.713
No	325 (93.12)	281 (93.36)	20 (90.91)	24 (92.31)	
Yes	24 (6.88)	20 (6.64)	2 (9.09)	2 (7.69)	
**DM** ^2^ **, n (%)**					**< 0.001**
No	264 (75.64)	238 (79.07)	18 (81.82)	8 (30.77)	
Yes	85 (24.36)	63 (20.93)	4 (18.18)	18 (69.23)	
**Chronic PD** ^3^ **, n (%)**					0.234
No	307 (87.97)	268 (89.04)	18 (81.82)	21 (80.77)	
Yes	42 (12.03)	33 (10.96)	4 (18.18)	5 (19.23)	
**Cort. User** ^4^ **, n (%)**					> 0.999
No	342 (97.99)	294 (97.67)	22 (100.00)	26 (100.00)	
Yes	7 (2.01)	7 (2.33)	0 (0.00)	0 (0.00)	
**Chronic KD** ^5^ **, n (%)**					0.191
No	341 (97.71)	295 (98.01)	22 (100.00)	24 (92.31)	
Yes	8 (2.29)	6 (1.99)	0 (0.00)	2 (7.69)	
**Comorbidities, n (%)**					**< 0.001**
No	182 (52.15)	173 (57.48)	7 (31.82)	2 (7.69)	
Yes	167 (47.85)	128 (42.52)	15 (68.18)	24 (92.31)	
**Address, n (%)**					0.685
Urban	191 (54.73)	162 (53.82)	13 (59.09)	16 (61.54)	
Village	158 (45.27)	139 (46.18)	9 (40.91)	10 (38.46)	
**Job, n (%)**					**< 0.001**
Employed	145 (41.67)	137 (45.51)	6 (28.57)	2 (7.69)	
Unemployed	203 (58.33)	164 (54.49)	15 (71.43)	24 (92.31)	
Unknown	1	0	1	0	
**Exposure, n (%)**					**0.014**
No	94 (26.93)	76 (25.25)	5 (22.73)	13 (50.00)	
Yes	107 (30.66)	90 (29.90)	7 (31.82)	10 (38.46)	
Unknown	148 (42.41)	135 (44.85)	10 (45.45)	3 (11.54)	

1: Tuberculosis, 2: Diabetes mellitus, 3: Chronic obstructive pulmonary disease, 4: Corticosteroids user, 5: Chronic kidney disease. * P < 0.05 was considered statistically significant (Bold items).

**Table 2:** Clinical and health system identification characteristics of the patients in the study

**Characteristics**	**Overall**, N = 349	**Cure**, N = 301	**Failure**, N = 22	**Death**, N = 26	**P-value**
**Main Symptoms, n (%)**					--
Cough	221 (63.32)	195 (64.78)	15 (68.18)	11 (42.31)	
Weight Loss	39 (11.17)	34 (11.30)	2 (9.09)	3 (11.54)	
Dyspnea	29 (8.31)	24 (7.97)	2 (9.09)	3 (11.54)	
Fever	26 (7.45)	18 (5.98)	1 (4.55)	7 (26.92)	
Anorexia	21 (6.02)	19 (6.31)	1 (4.55)	1 (3.85)	
Fatigue	10 (2.87)	8 (2.66)	1 (4.55)	1 (3.85)	
Chest pain	2 (0.57)	2 (0.66)	0 (0.00)	0 (0.00)	
**Report place, n (%)**					--
Healthcare Facilities^1^	125 (35.82)	114 (37.87)	6 (27.27)	5 (19.23)	
(Private) Clinics	95 (27.22)	82 (27.24)	10 (45.45)	3 (11.54)	
Government Hospitals	95 (27.22)	76 (25.25)	3 (13.64)	16 (61.54)	
Prisons	29 (8.31)	25 (8.31)	3 (13.64)	1 (3.85)	
Private Hospitals	5 (1.43)	4 (1.33)	0 (0.00)	1 (3.85)	
**Detector person, n (%)**					--
General Practitioner	99 (28.37)	88 (29.24)	8 (36.36)	3 (11.54)	
Internist	72 (20.63)	59 (19.60)	7 (31.82)	6 (23.08)	
Pulmonologist	63 (18.05)	55 (18.27)	1 (4.55)	7 (26.92)	
Infectious Disease Specialist	60 (17.19)	48 (15.95)	5 (22.73)	7 (26.92)	
Health Worker	55 (15.76)	51 (16.94)	1 (4.55)	3 (11.54)	
**Health system delay: month, n (%)**					0.230
< 1	109 (31.23)	91 (30.23)	7 (31.82)	11 (42.31)	
1-3	168 (48.14)	148 (49.17)	9 (40.91)	11 (42.31)	
3-6	39 (11.17)	37 (12.29)	1 (4.55)	1 (3.85)	
> 6	33 (9.46)	25 (8.31)	5 (22.73)	3 (11.54)	

1: Healthcare Facilities are environmental units providing health services and have the main task of guiding, supervising and supporting health units including “Urban Health Center - Rural Health Center – Rural Health House”.

**Table 3:** Univariable analysis for evaluation of predictors of unsuccessful treatment outcome in Tuberculosis patients

**Variables/evaluated item** ^1^	**Unadjusted OR (95% CI)**	**Standard errors**	**Statistics**	**P value** ^*^
**Sex/Female**	0.648 (0.337-1.207)	0.323	-1.344	0.179
**Age**	1.039 (1.022-1.058)	0.009	4.471	**< 0.001**
**Education/Low Education**	3.008 (1.255-8.932)	0.49	2.245	**0.024**
**Drug use/Yes**	2.337 (1.247-4.357)	0.318	2.673	**0.007**
**TB** ^2^ ** History/Yes**	1.277 (0.359-3.57)	0.571	0.428	0.668
**DM** ^3^ **/Yes**	3.197 (1.69-6.018)	0.322	3.604	**< 0.001**
**Comorbidities/Yes**	5.857 (2.857-13.29)	0.388	4.559	**< 0.001**
**Job/Unemployed**	4.072 (1.934-9.656)	0.405	3.467	**< 0.001**

1: The listed items have been compared with another item of their category, namely reference; for example, female item is compared to male item; 2: Tuberculosis, 3: Diabetes mellitus. * P < 0.05 was considered statistically significant (Bold items).

**Table 4:** Multivariable analysis for evaluation of predictors of unsuccessful treatment outcome in Tuberculosis patients

**Variables/evaluated item** ^1^	**Adj-OR** ^2^ ** (95% CI)**	**Standard errors**	**Statistics**	**P value** ^*^
**Sex/Female**	0.307 (0.135- 0.672)	0.406	-2.9	**0.004**
**Age**	1.014 (0.989-1.039)	0.0124	1.11	0.266
**Education/Low Education**	1.439 (0.432-5.69)	0.642	0.567	0.571
**Drug use/Yes**	0.842 (0.364-1.88)	0.417	-0.411	0.681
**TB History/Yes**	1.3 (0.322-4.319)	0.65	0.404	0.686
**DM/Yes**	1.105 (0.492-2.503)	0.412	0.242	0.809
**Comorbidities/Yes**	4.151 (1.567-11.64)	0.508	2.8	**0.005**
**Job/Unemployed**	3.312 (1.339-8.891)	0.478	2.5	**0.012**

1: The listed items have been compared with another item of their category, namely reference; for example, female item is compared to male item; 2: Adjusted OR. * P < 0.05 was considered statistically significant (Bold items).

## Discussion

TB is still one of the main infectious diseases (5). Several factors can shift the TB infection to the active form. In fact, the role of factors that weaken the immune system, such as DM, smoking and drug addiction, and malnutrition is important in the development of this disease and should be evaluated continuously (4, 11, 15, 16). Similar to other studies, our investigation showed that the two groups of people are more susceptible to the disease: the elderly, who have a weaker immune system, and those in the active group, who have mostly underlying diseases and are more socially active (4, 7, 15, 17).

Another important finding in the present study suggested that indices representing the quality of life of patients such as drug use, low education, and unemployment were associated with higher chances of developing the disease. For years, TB was a disease associated with poverty (7, 13, 15). Khaliq et al. found that more cases of TB were more prevalent in poor cases with characteristics such as unemployment, lack of education, and migration (15). However, further research is needed to verify this claim. In this regard, smoking and drug addiction, which are more prevalent in these communities, can negatively affect the function of pulmonary macrophages, epithelial cells, and natural killer cells, resulting in more bacteria colonization and the development of TB (17, 18). The results of similar studies indicate that TB may be reduced by increasing knowledge about the ways of transmitting infectious diseases and by educating societies (7, 17, 19).

According to WHO reports, cough for more than two weeks followed by fever or weight loss can be the most important and common symptoms of the disease. They recommend that general practitioners (GPs) or other health professionals should always consider these symptoms in the first lines of diagnosis in (rural) health centers, even in an area with a low prevalence of TB (17, 20). Bojovic et al. proposed that health system delay is related to the insufficient GPs experience in primary healthcare institutions (14). To prevent health system delays, GPs and others on the frontlines of the fight against TB must receive proper training in TB diagnosis. The rates of successful treatment in this study were similar to the global standard (WHO international target = 85%) and other studies (4, 9). Consistent with several previous studies, a significant percentage of treatment failures occurred in elderly patients, with low education, unemployment, and underlying diseases such as DM (7, 19). As a result, the disease will be better controlled with the correct education to the community (reducing the person delay system) as well as the medical staff (reducing the health system delay).

This study suffers from some limitations. First, the fact that samples were recruited from one province of Iran may prevent us from generalizing our results; although we tried to examine the cases from a variety of cultural and geographical locations to cover this. Secondly, the quality of the study was influenced by its retrospective nature. One of the important factors in tuberculosis, which is directly related to the treatment of the disease, is the HIV infection status. This factor was not investigated due to the lack of ability to verify the accuracy of data registration and also its incompleteness. Several national cohort studies focusing on these existing risk factors can cover these two limitations and provide more accurate results.

It was found that aging, underlying diseases, and cultural poverty are still at the top of the risk factors for active TB and/or for the treatment outcome. Considering these risk factors and continuous proper training of medical staff about the symptoms of various types of TB diseases can reduce the time of diagnosis, increase the detection rate of TB patients in each area, and prevent the further spread of the disease.
